# 576 Head and Neck Burn Injuries: A Nationwide Analysis in Pediatric and Adult Populations

**DOI:** 10.1093/jbcr/irae036.210

**Published:** 2024-04-17

**Authors:** Daniel Najafali, Michael Pozin, Justin M Camacho, Quincy K Tran

**Affiliations:** Carle Illinois College of Medicine, Urbana, IL; Drexel university college of medicine, New york, NY; University of Maryland School of Medicine, Baltimore, MD; Carle Illinois College of Medicine, Urbana, IL; Drexel university college of medicine, New york, NY; University of Maryland School of Medicine, Baltimore, MD; Carle Illinois College of Medicine, Urbana, IL; Drexel university college of medicine, New york, NY; University of Maryland School of Medicine, Baltimore, MD; Carle Illinois College of Medicine, Urbana, IL; Drexel university college of medicine, New york, NY; University of Maryland School of Medicine, Baltimore, MD

## Abstract

**Introduction:**

Burns to the head and neck impact a patient’s aesthetics in the most direct way and in severe cases impair function and quality of life. The purpose of this study is to compare outcomes between adult and pediatric patients sustaining burns to the head and neck region.

**Methods:**

Head and neck burn encounters were isolated from the ABA Burn Care Quality Platform between January 2008 and December 2018. Cohorts were separated into two based on patient's age (≤14 years and 15+ years). Descriptive statistics summarized patient demographics and clinical characteristics. Multivariable regression analysis determined predictors associated with any complications, increased lengths of stay, and mortality.

**Results:**

The analysis identified 52,272 individuals (mean age=34 years) sustaining a burn to the head and neck, of which 12,663 (24%) were pediatric cases (mean age=4 years) and 71% were male. The majority of adult and pediatric patients were White, 64% and 38% respectively. The top 3 etiologies for adults were: 1) flame, 2) scald, and 3) flash burns. For pediatric cases they were: 1) scald, 2) flame, and 3) contact burns. Burn severity as indicated by TBSA had a median [IQR] of 7% [3-17%] for adults and 6% [3-12%] for pediatric patients (p< 0.001). The mean (SD) percent of the burn concentrated on the head and neck was 42% (34%). Any complication was significantly associated with pediatric cases (OR 1.60, 95%CI 1.48-1.75), identifying as Hispanic or Latino (OR 3.80, 95%CI 1.63-8.44), and increased burn surface area to the head and neck (OR 1.01, 95%CI 1.00-1.01). Black patients (OR 1.90, 95%CI 1.60-2.20), patients with inhalation injury (OR 3.10, 95%CI 1.50-6.20), and greater TBSA (OR 1.10, 95%CI 1.10-1.10) had higher odds of mortality.

**Conclusions:**

Pediatric patients recovered faster and were more likely to survive their burn injuries to the head and neck. Adverse outcomes were observed to affect marginalized racial/ethnic communities. The existing disparities require increased attention and efforts should be made to determine any potential environmental or cultural factors that may predispose groups to more severe head and neck burn.

**Applicability of Research to Practice:**

These findings can help guide and educate clinicians in regard to head and neck burns as well as the existing disparities and epidemiological trends implicated.

